# Conceptualising the Factors Affecting Retention in Care of Patients on Antiretroviral Treatment in Kabwe District, Zambia, Using the Ecological Framework

**DOI:** 10.1155/2017/7356362

**Published:** 2017-11-09

**Authors:** Ferdinand C. Mukumbang, Joyce Chali Mwale, Brian van Wyk

**Affiliations:** School of Public Health, University of the Western Cape, Cape Town, South Africa

## Abstract

**Background:**

HIV remains a major public health challenge in Zambia. The roll-out of antiretroviral treatment (ART) has engendered new challenges in retention in care.

**Objective:**

To conceptualise the factors affecting retention in care of ART patients at three primary healthcare facilities using the ecological framework.

**Method:**

Qualitative data were collected through in-depth interviews with 45 ART patients and three focus group discussions with 20 healthcare providers from three primary healthcare facilities in Kabwe district, Zambia, and subjected to thematic content analysis.

**Results:**

Individual level barriers to retention in care included side effects, gaining weight, belief in faith healing, and use of herbal remedies and alcohol. Interpersonal barriers such as stigma and nondisclosure of HIV status were reported. At the institutional level, inadequate space in the clinic, long waiting times, long travel distances, and shortage of third-line drugs presented barriers to retention in care. Food shortages and patient mobility were reported as community barriers to retention in care.

**Conclusion:**

The ecological framework conceptualises the complex and dynamic factors affecting retention in ART care and highlights the need for multifaceted interventions that combine health education, disease management, and opportunities for income generation in a socially responsive and accountable environment.

## 1. Introduction

HIV/AIDS remains a major public health problem in sub-Saharan Africa, causing the death of millions of adults in their prime, disrupting and impoverishing families and turning millions of children into orphans [1]. At the end of 2016, 1.2 million people were living with HIV in Zambia, of whom a million were adults aged 15–49 years and 540,000 were women [2]. The Zambian Ministry of Health responded to the HIV epidemic by developing policies and treatment guidelines for the testing and treatment of people living with HIV (PLHIV). Consequently, there has been a rapid scale-up of treatment, with 671,066 PLHIV initiated on antiretroviral treatment (ART) at the end of 2014. The rapid scale-up to initiate PLHIV on ART, nevertheless, is accompanied by challenges of suboptimal retention in care and poor medication adherence [3], especially in the context of fragile healthcare systems, which characterises most low- and middle-income countries [4].

Although the number of people receiving ART continues to rise in Africa, it is estimated that approximately three-quarters of adults living with HIV in sub-Saharan Africa have not achieved viral suppression [5]. Many studies have shown that the proportion of patients that remain in care following ART initiation is low and retention in care remains a challenge in many countries with a high burden of HIV/AIDS [6]. A review of 33 patient cohorts taking ART in 13 African countries suggested that only 60% of patients remained in ART programmes after two years, with loss to follow-up (patients with unknown outcomes) accounting for 56% of all attrition [7]. Although we could not obtain actual retention-in-care rates, the Zambia Population-Based HIV Impact Assessment (ZAMPHIA) reported viral load suppression among PLHIV aged 15 to 59 years in Zambia at 59.8%–61.3% and 57.5% among females and males, respectively, by the end of 2016 [8]. These low viral load rates are suggestive of equally low retention-in-care rates.

Nonadherence to ART is identified as the most common reason for treatment failure [9]. Thus, retaining patients on ART is of public health importance as it is directly linked to access to medication and medication adherence [10]. Nonadherence to ART poses major challenges to reducing new HIV infections, addressing health disparities, and improving health outcomes [11]. Retention in care—the ability of PLHIV to adhere to critical aspects of care, such as attending regular follow-up appointments, scheduled laboratory tests, and other monitoring activities as prescribed by the healthcare provider [12]—is, therefore, an important aspect of ART programmes. In this study, retention in care was defined as patients attending their last scheduled follow-up appointment.

Issues related to the retention in care of patients on ART have been previously investigated in many regions in low- to middle-income countries [14] including Zambia. It has been observed that factors influencing retention-in-care behaviours are similar to those that influence adherence to ART [11]. If patients are not retained in care, it becomes a challenge for these patients to receive medications regularly, and thus retention in care is a critical mediator to medication adherence. Stricker and colleagues suggest that “retention in care and adherence to ART are critical elements of HIV care interventions and are closely associated with optimal individual and public health outcomes” [15]. Barriers to retention in care and adherence to medication could lead to increased morbidity and mortality in the patients through suboptimal viral suppression, increased risk of drug resistance, and increased risk of HIV transmission [16]. The spillover effect is a loss of or reduction in individual income, potentially translating to lower economic productivity at the country level and more expenditure of the health system through longer hospital stays and regimen switch to second- and third-line ART drugs [15].

Various models have also been applied to classify the factors affecting retention in ART care and adherence to treatment. For instance, Holtzman and colleagues used Andersen's Behavioural Model to map patient-identified barriers and facilitators to retention in HIV care and antiretroviral therapy adherence [17]. Other studies have classified these factors under various headings such as individual-related factors, medication-related factors, health systems related factors, socioeconomic factors, and sociocultural factors [18]. The goal of conceptualising these factors within a framework is to make sense of the complex and dynamic factors affecting retention in care of patients on ART. Nevertheless, the authors did not find any studies framing these factors on the ecological framework, a public health focus towards understanding the dynamic interrelations among various personal and environmental factors affecting retention in ART care. Therefore, our objective was to conceptualise the factors affecting retention in care of patients receiving ART at three primary health facilities in Kabwe district in Zambia using the ecological framework. Mapping the factors influencing poor retention in ART care using the ecological framework could provide a useful model for understanding and classifying these challenges, an important step towards a comprehensive and preferential solution approach in the context of resource constraints.

### 1.1. Conceptual Model

**Figure 1:**
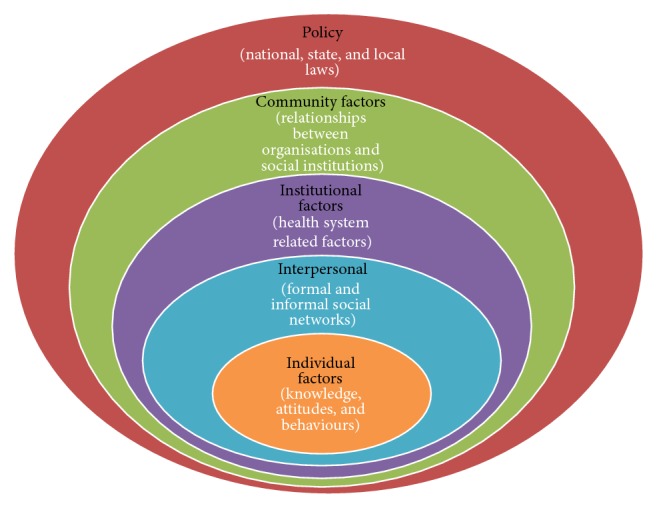
The ecological model [13].

The study was guided by the ecological framework, a theory-based framework for understanding the multifaceted and interactive effects of personal and environmental factors that determine behaviours. We thought that this model would be useful to understand the interaction of personal, health system, and environmental factors that affect the retention in care of PLHIV on ART in Kabwe district.  Figure 1 indicates the different components (levels) that constitute the socioecological model.

The ecological perspective epidemiology emphasises the interaction between, and interdependence of, factors within and across all levels of a health problem. Two important perspectives that guide the ecological perspective are that behaviour affects and is affected by multiple levels of influence and that individual behaviour shapes and is shaped by the social environment.

As illustrated in Figure 1, these levels are (1) intrapersonal or individual factors, (2) interpersonal factors, (3) institutional or organisational factors, (4) community factors, and (5) public policy factors [13]. The ecological framework is based on evidence that no single factor can explain health seeking behaviours or a health related problem and highlights the importance of people's interactions with their physical and sociocultural environments in determining their health behaviours (or perpetuating the health problem). Therefore, the ecological framework recognises multiple levels of factors that influence a person's health behaviours. In this way, the framework considers the interaction between factors at the different levels as being of equal importance. The model assumes that appropriate changes in the social environment will produce changes in individuals and that the support of individuals in the population is essential for implementing environmental changes [13].

## 2. Research Method

### 2.1. Setting

The study was conducted in Kabwe district of the central province of Zambia. Kabwe district is one of the six districts in the central province of Zambia. Kabwe district is approximately 139 km from the capital city Lusaka and covers a surface area of 1,577 km^2^. According to the Census of Population and Housing of 2010, Kabwe has an estimated population of 210,979 inhabitants with a population density of 128.7 persons per km^2^. The population consists of 48.8% males and 51.2% females and the average annual rate of population growth is estimated at 1.4% [19]. According to the MoH data of 2014, the district's population has increased to 217,843. Kabwe district is a transit town with huge traffic of people moving through the Great North road to the Copperbelt and northern part of Zambia from Lusaka and vice versa. The district is comprised of multilingual ethnic groups, with Bemba being the most widely spoken local language. Economically, Kabwe district has high (64%) poverty levels due to high unemployment rates brought about by the closure of factories and mines in 1995. Economic activities are mainly informal, though a few people are formally employed.

## 3. Design

A descriptive qualitative study design was used. According to V. Lambert and C. E. Lambert [20], it allows for the “comprehensive summarisation, in everyday terms, of specific events experienced by individuals or groups of individuals.” The authors suggest that this research design should be used when a straightforward description of a phenomenon is desired. A description of the different factors allowed us to classify them within the different levels of the ecological model.

### 3.1. Sampling

A sample of 65 participants participated in the study representing two groups: healthcare providers and patients. Using facility ART registers, an electronic smart care system, a pharmacy logbook, and patient locator forms, 45 patients—36 who were on ART at the time of the study and nine who had discontinued treatment (defaulted)—were purposively selected. Fifteen participants were selected from each ART site. Participants who were not able to come to the clinic were contacted and interviewed at their homes with the aid of patient locator forms and the adherence support workers. At each site (clinic), participants were recruited with the help of healthcare professionals. The characteristics of the study participants are displayed in Table 1.

The healthcare providers were also recruited purposively to provide an alternative source of information with regard to the factors influencing retention in care.  Table 2 displays the number and distribution of healthcare providers included in the study.

### 3.2. Data Collection

Two data collection methods were employed: individual in-depth interviews with patients using semistructured (open-ended) questions and focus group discussions with health workers. Semistructured interviews (using interview guides) allowed us to explore why patients on ART discontinue their clinic visits. Twenty health workers participated in the focus group discussions. Three focus group discussions were held, one at each site. Nine nurses, five clinical officers, and six adherence workers participated in total.

The interviews and focus group discussions were conducted in Bemba, the local language, which enabled the participants to clearly understand the questions and express themselves fluently. The interviews and the focus group discussions were audio-recorded with the participants' permission. To ensure anonymity of the study participants, the participants in the focus group discussions were given numbers during the sessions by which to identify them. The recorded sessions (interviews and group discussions) were transcribed verbatim and translated from Bemba to English by the second author who is fluent in both languages. The translations were checked by a research assistant who is also fluent in both languages. Any disparities in the meanings of words or phrases were addressed through the process of reconciliation between the second author and the research assistant.

## 4. Ethical Considerations

Ethics clearance was granted by the University of the Western Cape's Senate Research Committee and the Zambian ERES Converge ethics committee. Permission to conduct the study in Kabwe was obtained from each of the participating health facilities, the Kabwe district Ministry of Health, and the District Health office.

Participation in the study was voluntary. The participants were required to sign a consent form to acknowledge their willingness to participate in the study. While conducting the focus group discussions, we asked the participants to use pseudonames that were provided at the start of each session to preserve the anonymity of the participants.

## 5. Data Analysis 

The data analysis was conducted in two steps: first, we coded the data according to a predetermined coding structure based on the ecological model (individual, interpersonal, institutional, and community factors). This step of the analysis was deductive because we were guided by the framework.

Secondly, we grouped related codes to form themes for each of the levels of the ecological model. A thematic data analysis approach was employed [21]. We (1) read the transcription notes several times to become familiar with the data, (2) identified key issues and recurrent themes emanating from the data, (3) coded and organised the data into categories, and (4) interpreted the data by fitting the identified factors within the ecological framework. The data were analysed with the aid of qualitative data analysis software, Atlas.ti version 8.0.

## 6. Rigour and Trustworthiness

Rigour and trustworthiness in qualitative research relate to credibility, dependability, transferability, and conformability of data and findings. The following actions were carried out to enforce rigour and trustworthiness in the study. (1) Triangulation of data collection methods (interviews and focus groups) and information sources (patients who are retained in care, patients who have defaulted, and healthcare workers) with regard to the sources and the findings with other studies improved the trustworthiness of the study. (2) Research team members checked the key points at the end of each interview to verify their understanding with participants. (3) An audit trail of all the documents and decision-making processes during the study was kept to ensure rigour. We followed the consolidated criteria for reporting qualitative research (COREQ) outlined by Long and colleagues [22] to conduct and report this study.

## 7. Results

**Figure 2:**
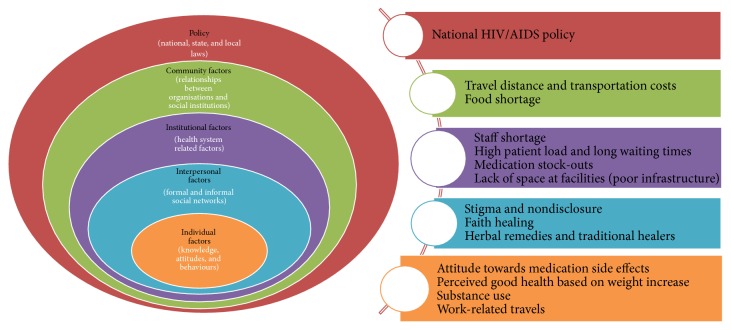
Summary of themes identified based on the components of the ecological model.

The study results are presented following the constructs of the ecological model (Figure 2).

### 7.1. Individual Factors

Factors identified at the intrapersonal or individual level relate to personal history and biological factors that influence the retention-in-care behaviours of the patient. Weight increase as a sign of improved health, medication side effects, and alcohol use were patient factors identified to influence retention-in-care behaviours. In addition, trust in faith healing and the use of other herbal remedies were identified as negative influences on retention in care.

#### 7.1.1. Weight Increase as a Sign of Good Health

Patients stop taking their medication after they notice an increase in body weight and start to look healthy because of taking their ART. Both the healthcare workers and the patients agreed that some patients stop taking their medication because they were feeling better and physically fit. Healthcare providers and patients reported on issues around improved health and retention in care.

*Some patients stop taking ARV medications when they notice an increase in weight and look healthy [because of taking medication]. They feel they can do away with the drugs especially when their CD4 count is high. This affects adherence and later impacts on long term retention*. (Healthcare worker)

*Some patients feel they are healed when they gain weight and start feeling better. There is this patient in my neighbourhood. He stopped taking his ARV medication after feeling well. He said there was no need for him to continue taking the ARV drugs*. (A 37-year-old woman)

#### 7.1.2. Side Effects

Both healthcare providers and patients identified medication side effects as contributing to poor retention in care. Side effects reported were nausea, vomiting, dizziness, and breast enlargement in male patients (gynaecomastia). These may affect patients' health service seeking behaviour and may lead to attrition from ART care because, for male patients, developing breasts interfered with their self-esteem.

*Observations made were that a few patients especially men may stop their ARV medications because of side effects like developing breasts (gynaecomastia). Other patients stop their medications because of nausea, vomiting and dizziness. Such are the patients who discourage the rest of the community to stop ARV drugs*. (Healthcare worker)

*Yes, they [medication] affected me. I have rashes on my body, and then I stopped the medication. I later felt sick, so I decided to go back to the hospital… I explained it to them [health care workers] and they changed the drugs*. (A 42-year-old woman)

#### 7.1.3. Alcohol Use

Excessive beer drinking was reported to affect patient retention in care and subsequently medication adherence. Some patients reported displaying heavy drinking behaviours after registering great improvement in their health. This is what a participant said:

*It has been now close to one year since I stopped my ARV treatment. I just stopped taking my ARV drugs because of beer drinking. When I got drunk, I used to forget to take my drugs and eventually I stopped my drugs*. (A 32-year-old woman)

### 7.2. Work-Related Travels

According to the participating health workers, patients who travel for work purposes from their hometown tend to miss clinic appointments and default ART and this contributes to nonadherence and poor retention. Two participants mentioned truck drivers who had defaulted treatment for months and then reappeared again for treatment. This is what they had to say:

*The other reason is I have seen many truck drivers. They tend to travel when given a contract. Then you find for months they will disappear. When they appear and you ask them why they disappeared, they say they went for work*. (Healthcare worker)

*Some patients miss treatment appointments when they travel to go and work in another town. I can attest to this. My neighbour is always traveling and when he comes back, he mentions not having taken his ARV drugs for a long time*. (Healthcare worker)

### 7.3. Interpersonal Factors

Factors classified under the interpersonal level relate to barriers that emanate as a result of patients sharing and interpersonal relationships with various persons or groups such as family, friends, intimate partners, and peers. Based on the data, the following themes could be identified under stigma and nondisclosure of HIV status.

#### 7.3.1. Stigma and Nondisclosure of HIV Status

HIV related stigma was reported as one of the reasons why some patients discontinued ARV treatment or missed treatment dosage. Healthcare providers reported that patients do not want to disclose their HIV status to either their marital partners or other members of their social network for fear of domestic violence, partner abandonment, or rejection by the community. As a result, patients opt to stop attending the clinic for ART services, which affects adherence. A patient also suggested that other patients hide their HIV status to start up and sustain relationships.

*Couples do not want to disclose their status to each other. You find that the wife is on ARVs without the husband knowing or the husband is on treatment without the wife knowing. Sometimes you find that the husband is taking drugs from the office without the partner being aware*. (Healthcare worker)

*Some patients hide from their relatives. Some patients who look healthy and want to get married hide and give up on treatment so that their partners do not get suspicious of their HIV status since they may lead to the end of the relationship. I have a sister from my mother's sister. She is now dead. She got married and hides her HIV status from her husband and stopped taking her ARV drugs*. (A 34-year-old woman)

Additionally, healthcare workers confirmed that stigma was still highly prevalent at individual and community levels.

*Stigma is still very high among the rich and those in business. Some of them send other people to pick their drugs for them. If the health care worker insists on seeing the patient, they would rather stop taking the ARV drugs for fear of being known. This is why they default and we admit them here when they come very sick and weak*. (Healthcare worker)

#### 7.3.2. Faith Healing

Religious beliefs and practices also may lead to patient attrition from ART care. Both patients and healthcare workers agree that some church pastors in their communities who conduct healing prayers sessions persuade patients to discontinue ART because they are healed.

*Some people go to some pastors who pray for them and tell them to stop taking their ARV drugs that they are healed. I have seen some people who discontinue treatment. One even died after stopping taking these drugs. He was my neighbour*. (A 42-year-old widow)

*Pastors who preach the gospel of faith healing convince many patients to discontinue ART. They feel healed when they are prayed for. They have faith in these pastors and they would definitely stop taking ARVs. Sometimes when they feel sick, that is when they reveal that I had stopped because I was told to stop because I was healed*. (Healthcare worker)

#### 7.3.3. Herbal Remedies

Use of herbal remedies to treat HIV related illnesses was reported by both patients and healthcare providers as a cause for discontinuation from the ART programme. Some patients were being told that herbal remedies could cure HIV and AIDS. This influences the health seeking behaviours of patients, leading to discontinuation of ART. The use of herbal medicines was also reported to have been easily accessed by some patients especially those from rural settings and that people have confidence in traditional healers.

*My neighbour used to drink these herbal remedies called Moringa from traditional healers because he was told it heals all diseases including HIV and AIDS. Unfortunately, he died*. (A 32-year-old woman)

*Some patients especially those from rural settings find these traditional medicines easily accessible and they have more confidence in tradition healers. Whatever they say is right. That component is also affecting patients' adherence to treatment and retention in care*. (Healthcare worker)

### 7.4. Institutional Related Factors

Institutional related factors speak to the health system factors and these factors relate to the way health services are organised and delivered and relates to access to the facility and to medication, the overall environment of the facility, the patient–provider relationship, and support services that are incorporated into care [8]. The following health system factors were identified: staff shortage, medication stock-out, congested facilities, long waiting times, and travel distance and cost of transportation.

#### 7.4.1. Staff Shortage

Healthcare providers and patients identified staff shortage as a factor influencing retention in care of patients on ART. This is because staff shortages lead to long queues at the clinic waiting area and, consequently, long waiting times. Because of this, patients get frustrated and tired of waiting to be seen.

*There are few health workers to attend to patients at ART centres. In most instances only one clinical officer or one nurse on full ART day working with two adherence support workers against a crowd of patients. Some patients get tired of waiting and go back home without being seen*. (A 36-year-old woman)

*There is only one person remaining at ART clinic to see patients. This clinical officer is overwhelmed with work and two days a week is too much for this person. Therefore, there are many compromises. If you saw today the queue that was there. He had to review the in-patients first and then outpatients. It's too much*. (Healthcare worker)

It was also reported that several patients discontinue treatment because of long waiting times at the ART clinics. Participants complained about long waiting times, which made them more likely to stop coming to the clinic to collect their drugs. Patients reported long waiting time of four to six hours at the clinics before receiving care services.

*The health workers are few. Sometimes there can be only one. The rest could be these volunteers. That is the reason why we even spend more time here*. (A 35-year-old widow)

*We take a long time here. Sometimes I spend about 4 hours and sometimes 5 hours depending on the time, they start the clinic. This is frustrating. You can even stop coming to this clinic to collect ARV drugs*. (A 37-year-old widow)

The healthcare providers corroborated that high patient load at ART centres leads to long waiting times and in some instances contributes to high defaulter rates.

*Actually, we have many patients at these clinics in relation to the health care workers. When we look at the trained adherence support counsellors they are not even enough to carter for all the patients*. (Healthcare worker)

#### 7.4.2. Stock-Out of Antiretroviral Medication

Healthcare workers and patients reported that ART clinics sometimes ran low on the ARV stocks, which affects the supply of medication received by the patients. Some participants reported receiving a one-month supply of ARV drugs when a three-month supply had been prescribed, while other participants reported frequent visits to the ART centres to be tiring especially when one does not have transport money. One participant reported a stock-out of third-line drugs, which are mostly found at referral hospitals, and patients had to travel to access treatment.

*If they say there is a shortage of ARV drugs, they write medication for three months but they will give you for one month then tell you to come and collect for remaining months. When you come, you just go straight to the pharmacy. However, this sometimes makes you tired especially where you have transport money*. (A 28-year-old man)

*We had a stock out sometime last year but all of this year we never had a stock out. We had many challenges for third line ARV drugs. We have some patients who are on third line treatment. Those on the third line are not accessing their drugs here despite being under this clinic. Them they go to Lusaka to access treatment. Because of this, they may not have transport to go to Lusaka for drugs. For example, we had to contribute to transport for one to go to Lusaka*. (Healthcare worker)

#### 7.4.3. Inadequate Space for ART Clinic

Healthcare providers reported limited space within the ART clinic as being a challenge towards the implementation of ART. This can have an impact on patient adherence and long-term retention. Participants reported using any free room or an empty office, which do not provide a waiting area for patients. In this case, confidentiality (privacy) during ART clinic is compromised because of overcrowding of patients.

*We do not have enough space at this clinic for ART patients. Instead, we use any room not in operation during the ART day, the office either for the sister in charge or the Male circumcision room. You can just see how this passage is congested. Because of this, even space for individual counselling has become a challenge as it leads to loss of patients in ART programme because confidentiality is compromised*. (Healthcare worker)

*Some patients tend to shun ART services where there is no confidentiality. We have many patients who are lost to follow up because of the same confidentiality due to lack of space for ART clinic*. (Healthcare worker)

### 7.5. Community Related Factors

The community, as relates to the ecological model, has three aspects: (1) mediating structures or face-to face primary groups, (2) relationships among organisations and groups within a defined area, and (3) a population with political entities [13]. Factors operative at these different levels of the community were considered. These factors include travel distance and cost of transportation, food shortage, and work-related mobility

#### 7.5.1. Travel Distance and Cost of Transportation

Although ART sites do not charge for HIV related services, travel distance and additional costs incurred traveling to ART centres may contribute to nonretention in the care of patients on ART programme more so for patients who cannot afford it. While some participants reported travel distance to ART centres as a barrier to accessing treatment, others also mentioned lack of money to pay for transport:

*Distance from home to the clinic is very long. I take one hour walking to this clinic when I am fast. I can even take an hour and 30 minutes if am not fast. Sometimes, if am not feeling well, I cannot walk this distance and if I do not have someone to come and collect the drugs for me, I miss the appointment date and come later when I feel better*. (A 42-year-old widow)

*Some patients who stay from distant places struggle to come to the clinic because of transport costs. Sometimes they would even resist to starting treatment or when they start they will pretend they will come back to the clinic because of distance*. (Healthcare worker)

*The other reason is about distance. Some places become impassable during the rainy season and it is not easy for them to access ARVs. You find in some areas bridges are swept away and this will cause some patients to default treatment. So, they can't find their way to the clinic*. (Healthcare worker)

#### 7.5.2. Food Shortage

Food insecurity at the household level may cause patients on ART to stop their medication. Both healthcare providers and patients reported a shortage of food in the household as a reason to default treatment, which may influence adherence and long-term retention.

*People who come from the poor settings where they have a shortage of food believe that they need to eat before they take ARV drugs. They will even tell you that I stopped because there was no one to buy food for me*. (Healthcare worker)

*Some patients stop treatment because they do not have food in their households. My neighbour stopped taking her ARV drugs because of a shortage of food. She was afraid to take the ARV drugs on an empty stomach*. (A 25-year-old woman)

## 8. Policy Factors

Based on the ecological model, aspects of policy are very important when examining the factors affecting the implementation of the public health intervention or programme. Nevertheless, we did not explore factors at this level with the study participants because we did not consider them knowledgeable enough to respond to the questions relating to the HIV/AIDS policy environment in the Kabwe region and the national HIV programme.

## 9. Discussion

Retention in care and adherence to medication behaviours have been previously described as complex and dynamic [3, 11]. The ecological model provides an explanatory framework to understand these factors that influence retention in care and adherence, without dispelling the complex nature of these behaviours.

Individual-related factors included side effects to ARV drugs, weight increase, and feeling better as a sign of good health. The findings of this study are consistent with the findings of other studies conducted in sub-Saharan Africa and elsewhere [23, 24]. Discontinuation of ART due to gynaecomastia and perception of good health could be indicative that patients are not properly educated on the importance of adherence to their medication even if they experience certain side effects or feel better. Roura et al. [23] argue that individuals' behaviour with regard to remaining in care is influenced by factors in the immediate environment such as support (or lack thereof) from significant others, work schedule, and disclosure. Patients may fail to adhere to medication in spite of their knowledge of the importance of adhering because their personal experience may be more persuasive than medical information.

Interpersonal factors influence the health of people and communities. They affect patient lifestyle by causing stress, social isolation, and fear of rejection [25]. When a patient's emotions, opinions, or behaviours are affected by others, adherence to medication and long-term retention are affected [26]. The social factors may include experiences, interpersonal relationships with marital partners, and family members that in turn affect individual health seeking behaviour and actions [25–27]. Social factors identified include stigma, use of traditional healers with herbal remedies, faith healing, and alcohol use and nondisclosure of HIV status. The results of this study are similar to those identified in qualitative studies in developing countries. For instance, a study conducted in Zambia by Musheke et al. [28] reported “feeling better,” use of herbal remedies, and faith healing as factors contributing to poor retention. A similar study conducted in Uganda by Mugisha et al. [24] also reported stigma and traditional healers as a considerable impediment to ART access, adherence, and long-term retention in care, which is at the individual, household, and community levels. The literature reveals that people living with HIV/AIDS fear to lose their social and emotional support by disclosing their HIV status to their spouses, social network, or other family members and fear of marriage breakdown in case of married people. Consequently, when social support is threatened by involuntary disclosure of HIV status, individuals abandon treatment [24].

Issues of long waiting times at health facilities due to high patient loads, staff shortages, and shortages of third-line ARV drugs were identified as health systems related factors affecting retention in care. Similar findings from the previous studies reported long waiting hours at health facilities due to high patient loads which affected patient access to treatment. Long waiting hours is among the key drivers of attrition for patients on ART [22, 29, 30]. It is recommended that adopting extended clinic hours to provide care after working hours or weekend could address the issue of clinic congestion especially for those patients that are employed [30]. Weekend clinics have been found to reduce clinic congestion and help retain patients in care.

Travel distance to ART health facilities and transport cost were reported to contribute to nonretention of patients on ART. This is mostly because ART centres are sparsely distributed within Kabwe district. In some instances, healthcare workers reported having donated transport fare to enable patients to access third-line treatment in Lusaka, approximately 139 km away from Kabwe. This study showed that distance to ART clinic is a barrier to both ARV adherence and access to care, which may affect long-term retention in care for patients in the ART programme. Similar studies from Uganda and Malawi also found the distance to ART centres as a barrier to patient retention [14, 24, 31].

The economic factors such as employment status, shortage of ARV drugs, and lack of food may influence patient retention in care. The living conditions and monitoring survey of 2006–2010 confirmed that 64% of Zambia's population falls below the national poverty line of US$1.08 per day and 90% of the labour force is in informal sector employment [19]. Even where people living with HIV are encouraged to continue accessing treatment, their health seeking behaviours are undermined by their fragile livelihoods. Concerns about the widespread poverty and food insecurity at the household level and their effect on long-term retention on ART have been previously identified [32, 33].

Stock-out of drugs was also reported by healthcare workers. Weak procurement and supply management systems in low-income countries which result in frequent shortages of ARV drugs and other essential commodities have been reported previously [1]. Similarly, many health systems are finding it difficult to ensure that there is an adequate and consistent supply of drugs and other supplies especially with the increasing number of patients initiating treatment [34].

Although healthcare workers during the focus group discussion reported intensive counselling of patients before and after ART initiation, the patients' knowledge and beliefs related to HIV and ART clearly showed that the information provided does not successfully dispel and correct the myths and misconceptions that some of the patients have. Lack of education and poor knowledge about ART and the HIV/AIDS can also lead to an inadequate understanding of the effectiveness of medications resulting in reduced adherence to treatment and poor retention of patients in care [35].

The study found factors that influence patient retention in care, which is diverse in nature and could be classified within the various spheres (levels) of the ecological model, namely, intrapersonal or individual factors, interpersonal factors, institutional or organisational factors, and community factors. This complex nature of the factors influencing retention in ART care makes it difficult to identify which of these factors is more important; even if it was possible to classify them in order of importance, the classification would have to be context-specific, thus increasing the complex nature of retention in care and adherence to medication behaviours. The study also demonstrates how individuals adjust their behaviour to their immediate social environment, which also affects their health seeking behaviour. According to Roura et al. [23], the processes through which other people's behaviours, advice, and beliefs determine health behaviour emerge as a main pathway for individual decision-making and reflect localised norms and levels of support within the individual's social networks. Roura et al. [23] also argued that the strength of social influence could severely undermine the capacity to follow through an intention to remain on treatment leading to dropping out from the treatment programme.

Following that, the ecological framework recognises multiple levels of factors that influence a person's health behaviours and the concept of reciprocal causation; addressing the challenges at one level of the framework could have a ripple effect on another area. For instance, if a patient receives effective intensive counselling and medication initiation education, he/she might refrain from using herbal treatment and other faith-based treatment options and focus on maintaining good adherence to ART. The motivation received from the counselling and educational sessions may encourage them to disclose their HIV status to their loved ones, which in turn enhances adherence based on received social support [18]. At the same time, the perceived social support also dispels perceived stigma, which enforces medication adherence. This explanation indicates a move between the intrapersonal and the interpersonal spheres of the ecological model.

In practice, addressing the community level requires taking into consideration institutional and public policy factors, as well as social networks and norms [36]. For instance, if the government of Zambia decides to open more ART service centres or implement community-based ART models to address the challenges related to long distances to the ART centres, they would have to consider the implications in terms of resources availability. In recent times, the use of community health workers to drive such initiatives as one strategy to address the growing shortage of health workers has been highlighted by various authors [37]. The application of this strategy would require the application of the policies that guide the use of community health workers. In the same way, the implementation of interventions like the community-based models would require buy-in and full participation from the community members for the intervention to work [38].

An ecological perspective shows the advantages of multilevel interventions that combine behavioural and environmental components. This resonates with the recent wave of community-based differentiated care models that are recommended for the improved management of PLHIV. Differentiated care models usually adopt a multifaceted approach towards enhancing patients' retention-in-care behaviours by embedding various strategies, including providing minimal clinical screening, quick ARV refills, health education, and adherence support within a single model [39]. Most of these models are recommended to take place in an environment that is easily accessible to the patients such as a local church, town hall, or NGO for easy medication pick-up (institutional or organisational factors). In addition to this, patients have the chance to receive continued counselling and education on the importance of remaining in care and adhering to medication (intrapersonal or personal). Group-based differentiated care models—having patients receive their care together to increase peer support among them and create an enabling treatment and care environment (intrapersonal)—could also improve their retention in care and medication behaviours. In addition, by separating the drug delivery and clinical care and reducing the intensity of the services, the care process is simplified for the providers and made much user-friendly (institutional or organisational factors). The implementation of such interventions is governed by policies at the provincial and national levels.

## 10. Limitations of the Study

While our study was focused on the factors affecting retention in ART care, we could only reach a few defaulters to participate in the study. Most of the patient participants were those currently retained in care. Limitations to the study were related to the possibility of the participants giving false responses due to the sensitive nature of the study. To reduce the chances of this occurring, the participants were reassured of confidentiality. The interviews were conducted in the Bemba local dialect and then translated to English. Translation from one language to another always introduces a chance for some meanings to be lost in the translation process. Losing meaning in translation was minimised through the process of data cleaning conducted by the first author who is fluent in both languages.

While adopting the ecological model to frame the findings of this study, we were aware of some of its limitations. For instance, it is criticised for falsely separating the social, economic, political, and cultural levels of interactions that might influence the other levels.

## 11. Recommendations

The findings of this study highlight the importance of strong community follow-up and patient support systems for all patients in the ART programme in Kabwe district [40]. There is a need to engage a wider range of partners such as community-based and faith-based organisations, local communities, and in particular people living with HIV to improve patient tracking and follow-up and, hence, retain them in care. To resolve the challenges related to the distance to the health facility and high transport cost, patients should be continually encouraged to access treatment at their nearest treatment centre.

## 12. Conclusions

The average retention rate in Kabwe district is about 65%; thus, 35% were considered to be lost to follow-up mainly due to social, economic, and structural factors. The findings of this study suggest that low retention rates are a result of complex and multidimensional factors. For easy identification and description, we have identified them following the ecological framework. These factors identified to influence retention of patients in ART programmes in Kabwe district are complex and highlight the need for multifaceted interventions that combine health education, disease management (by patient and health services), and opportunities for income generation in a socially responsive and accountable environment.

## Figures and Tables

**Table 1:** Characteristics of ART patients who participated in the study.

Characteristics	Frequency (*N* = 45)
Sex	
Female	25
Male	20
Age (in years)	
18–24	3
25–34	21
35–44	14
45 and above	7
Marital status	
Single	8
Married	23
Divorced/separated	4
Widow(er)	10
Level of education	
None	3
Primary school	29
Secondary school	13
Tertiary	0
Source of livelihood	
Formal employment	2
Self-employed	32
Unemployed	7
Dependents	4

**Table 2:** Healthcare workers in three FGDs.

Category of health workers	Katondo Clinic	Ngungu Clinic	Makululu Clinic	Total
Nurses	4	2	3	9
Clinical officers	1	3	1	5
Adherence workers	1	2	3	6

Total	6	7	7	20
